# Supplementary material for: The Liver Microbiome Is Implicated in Cancer Prognosis and Modulated by Alcohol and Hepatitis B
Source: Cancers (Basel). 2020 Jun 21;12(6):1642. doi: 10.3390/cancers12061642 (PMC7353057; doi:10.3390/cancers12061642)
Supplement: Supplementary file 1 [file cancers-12-01642-s001.pdf]

Supplementary Materials: The Liver Microbiome Is Implicated in Cancer Prognosis and Modulated by Alcohol and Hepatitis B

Jaideep Chakladar, Lindsay M. Wong, Selena Z. Kuo, Wei Tse Li, Michael Andrew Yu, Eric Y. Chang, Xiao Qi Wang and Weg M. Ongkeko

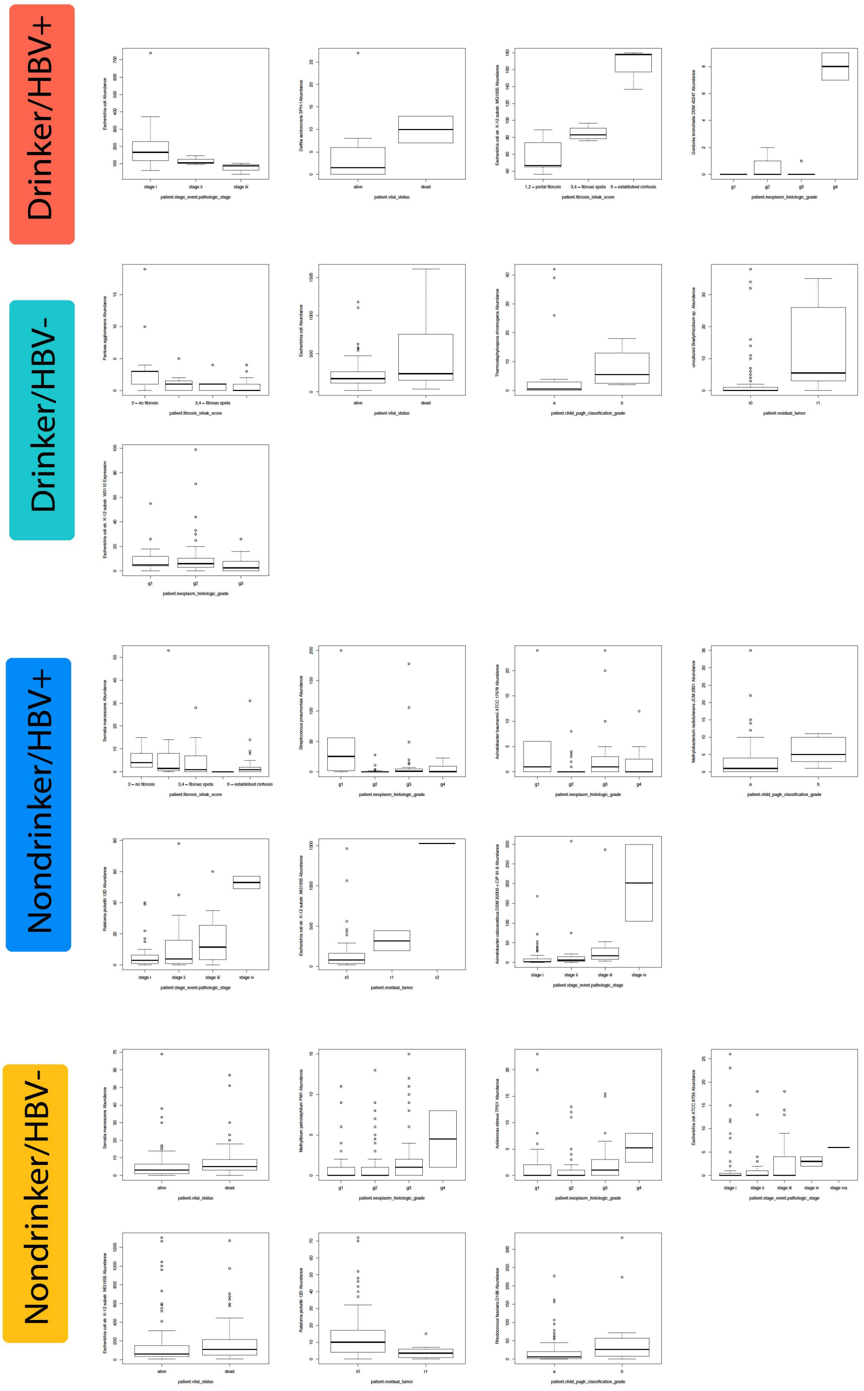

Figure S1: Kruskal wallis box plots for clinical variable correlations. A visualization of the top kruskal-wallis box plots comparing clinical variables to microbial abundance for each of the four comparisons. Clinical variables plots correspond to the *p*-values plotted in Figure 4.

© 2020 by the authors. Licensee MDPI, Basel, Switzerland. This article is an open access article distributed under the terms and conditions of the Creative Commons Attribution (CC BY) license (http://creativecommons.org/licenses/by/4.0/).
